# The efficacy and safety of combination of PD-1 and CTLA-4 inhibitors: a meta-analysis

**DOI:** 10.1186/s40164-019-0150-0

**Published:** 2019-10-25

**Authors:** Kongju Wu, Ming Yi, Shuang Qin, Qian Chu, Xinhua Zheng, Kongming Wu

**Affiliations:** 1 Department of Clinical Medicine, Medical School of Pingdingshan University, Pingdingshan, Henan 467000 People’s Republic of China; 2 Department of Oncology, Tongji Hospital of Tongji Medical College, Huazhong University of Science and Technology, Wuhan, 430030 China

**Keywords:** Immunotherapy, PD-1, PD-L1, CTLA-4, Immune checkpoint inhibitor, Combination therapy, Meta-analysis, Systematic review

## Abstract

**Background:**

Recently, a series of clinical trials showed that combination of anti-programmed cell death-1 (α-PD-1) and anti-cytotoxic T-lymphocyte-associated protein 4 (α-CTLA-4) could effectively eliminate tumor. However, in comparison with widely adopted mono-immune checkpoint inhibitors, chemotherapy, and targeted therapy, the advantage of combination therapy of α-PD-1 and α-CTLA-4 in response rate and prognosis is controversial especially considering probably increased treatment related adverse event. Thus, we conducted this meta-analysis to explore the efficacy and safety of combination treatment of α-PD-1 and α-CTLA-4.

**Methods:**

This meta-analysis involved 8 clinical trials. In most trials, the primary endpoint was objective response rate (ORR). Thus we calculated risk ratio (RR) and 95% confidence interval (CI) to compare ORR of patients undergoing different treatment strategies. Moreover, the co-primary endpoints in few trials included progression-free survival and overall survival. Hazard ratio (HR) with 95% CI were employed to weigh the influence of different treatments on prognosis of patients. Subgroup analysis was conducted in patients with high and low expression of PD-L1. Lastly, the safety of combination therapy was evaluated by comparing treatment related adverse events among various treatment groups.

**Results:**

Our results showed that ORR was significantly higher in patients receiving α-PD-1 plus α-CTLA-4 compared with α-PD-1 (RR 1.31, 95% CI 1.16–1.48) or α-CTLA-4 monotherapy (RR 2.11, 95% CI 1.84–2.43), chemotherapy and targeted therapy (RR 1.41, 95% CI 1.26–1.58). α-PD-1 plus α-CTLA-4 treated patients had a great advantage on monotherapy, chemotherapy and targeted therapy treated patients in PFS. Notably, no significant alteration in total adverse event rate was observed in α-PD-1 plus α-CTLA-4 treated patients. Results of subgroup analysis showed that combination therapy could enhance anti-tumor response in comparison with other treatments, especially for low PD-L1 expression patients undergoing nivolumab treatment (ORR: RR 1.35, 95% CI 1.11–1.65).

**Conclusion:**

Combination treatment of α-PD-1 and α-CTLA-4 is a feasible strategy with enhanced efficacy and acceptable adverse event. Moreover, for some low PD-L1 expression patients, α-CTLA-4 might decrease the risk of resistance to α-PD-1 and demonstrate the synergistic anti-tumor effect.

## Background

Cancer-immunity cycle model was established in 2013 to describe a series of stepwise events regulating anti-tumor immune response [1]. In this model, immune checkpoints act as inhibitory modulators and help cancer cell escape immune surveillance [2, 3]. As a vital immune checkpoint molecule, cytotoxic T-lymphocyte-associated protein 4 (CTLA-4) is constitutively expressed by regulatory T cells (Tregs) but transiently expressed by conventional T cells post activation [4–6]. Apart from T cell receptor (TCR) recognizing antigen peptide-major histocompatibility complex, CD28 binding to CD80 or CD86 is an essential co-stimulatory signal for T cells activation. CTLA-4 is a competitive antagonist for CD28-CD80/86 binding which further impedes priming and activation of T cells [7].

Similarly to CTLA-4/CD28 pathway, programmed cell death-1/programmed cell death ligand-1 (PD-1/PD-L1) axis is another important regulatory signal determining immune status [8]. PD-1 is mainly expressed on activated T cell which could transduct extracellular signal (PD-L1) [9]. Intracellular domains of PD-1 subsequently inhibit Ras-Raf-MEK-ERK and PI3K-AKT pathways by which extracellular PD-L1 undermines cytotoxicity activity of T cell [10, 11].

In theory, anti-PD-1 (α-PD-1) plus anti-CTLA-4 (α-CTLA-4) treatment simultaneously block two inhibitory signaling pathways of anti-tumor immune response [12]. However, in some clinical trials, no significant advantage was observed in therapeutic effect parameters such as objective response rate (ORR), progression-free survival (PFS), and overall survival (OS) for patients undergoing α-PD-1 plus α-CTLA-4 treatment, especially in comparison with α-PD-1 monotherapy treated patients [13]. Besides, combination therapy might increase the risk of treatment related adverse event, causing treatment discontinuation [14]. Therefore, the combination therapy might not absolutely bring benefit to patients.

To comprehensively compare the efficacy and safety of combination therapy of α-PD-1 and α-CTLA-4 with monotherapy, chemotherapy, and targeted therapy, we reviewed the relevant clinical trials and conducted this meta-analysis. Moreover, given the crucial role of PD-L1 expression in immune checkpoint inhibitor treatment, we performed a subgroup analysis to evaluate efficacy difference among different treatments in the context of high or low PD-L1 expression [2].

## Methods

### Study design and systematic review protocol

This meta-analysis was designed based on Preferred Reporting Items for Systematic Reviews and Meta-Analyses (PRISMA) statement [15].

### Participants, interventions, comparators

Randomized controlled trials included in the meta-analysis all consisted of one treatment arm (α-PD-1 plus α-CTLA-4) and one or two control arms such as α-PD-1 or α-CTLA-4 monotherapy, chemotherapy, and targeted therapy. ORR, PFS, and OS were primary parameters to evaluate efficacy of treatment. Response Evaluation Criteria in Solid Tumors [RECIST] 1.1 was adopted to measure treatment outcome. The safety of treatments was estimated by probability of any grade and 3–4 grade adverse event. The assessment of adverse event was according to National Cancer Institute Common Terminology Criteria for Adverse Events (version 4.0).

### Search strategy

We searched PubMed and Cochrane Library databases for eligible studies on June 23 2019 with search terms and Boolean operators as following: “(((Ipilimumab) OR Tremelimumab)) AND (((((Atezolizumab) OR Avelumab) OR Durvalumab) OR Nivolumab) OR Pembrolizumab)”.

### Data sources, studies selections and data extraction

All studies included in the meta-analysis met the following criteria: (1) randomized controlled trial; (2) efficacy and/or safety of α-PD-1 plus α-CTLA-4 therapy was investigated; (3) patients in control arm received other treatments except for combination treatment mentioned above; (4) efficacy and safety data were available in the paper. Studies were excluded according to the standards as: (1) non-randomized controlled trial; (2) efficacy and safety data were not available in the paper; (3) patients in control arm underwent combination treatment as well, aiming to explore the influence of dose on treatment effect; (4) article contained updated data in the followed-up paper.

Two authors (Ming Yi and Shuang Qin) independently selected studies met the inclusion criteria. All screened studies were filtered by title and abstract firstly. Then, uncertain studies were assessed by full-text review. For all studies included in meta-analysis, we extracted data including study name, first author name, cancer type, treatment arm, control arms, number of patients assigned into every arm, efficacy data, and safety parameters. For few studies without available Hazard Ratio (HR) and 95% confidence interval (CI), HR value was estimated from Kaplan–Meier curve by Engauge-Digitizer software.

The Cochrane Collaboration’s tool for assessing risk of bias was employed to assess each involved study [16]. Selection bias, performance bias, detection bias, attrition bias, as well as reporting bias were evaluated.

### Data analysis

Comparison of efficacy between combination therapy and other treatments was conducted by RR of ORR, HR of PFS and OS. Safety of treatment was evaluated by RR of adverse event. Heterogeneity among treatment groups was assessed by Chi square-based Q statistic. If I^2^ > 50% or *p *< 0.05, random-effect model was adopted [17]. Otherwise, fixed-effect model was employed. A funnel plot with 10 studies or less is often misinterpreted, so we did not assessed publication bias [18]. All data were performed by Stata software (version 12.0).

## Results

### Flow diagram, study selection, and characteristics

**Fig. 1:**
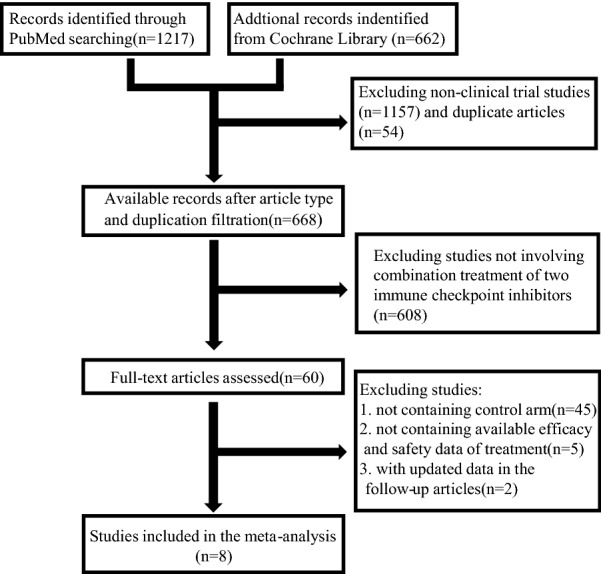
Flow diagram of literature retrieval process

As the literature retrieving process shown in Fig. 1, we searched PubMed and Cochrane Library databases and obtained total 668 studies after filtration of replicated document and article type. By reviewing title, abstract, and full-text, we finally selected 8 studies for meta-analysis which included 1730 combination therapy treated patients, 855 nivolumab (α-PD-1) treated patients, 362 ipilimumab (α-CTLA-4) treated patients, 546 sunitinib (targeted therapy) treated patients, and 583 chemotherapy treated patients. All included patients comprised 85 sarcoma patients, 160 esophagogastric cancer patients, 1153 melanoma patients, 1096 renal-cell carcinoma patients, 20 glioblastoma patients, and 1562 non-small cell lung cancer patients. Characteristics of eligible studies were shown in Table 1.

**Table 1:** Characteristics of clinical trials included in the meta-analysis

Study (phase)	First author	Cancer type	Treatment arm (No. of patients)	Control arm 1 (No. of patients)	Control arm 2 (No. of patients)	Refs.
Alliance A091401 Phase 2	D’Angelo SP	Sarcoma	Nivolumab plus Ipilimumab (42)	Nivolumab (43)		[29]
CheckMate 032 Phase 1/2	Janjigian YY	Esophagogastric Cancer	Nivolumab plus Ipilimumab (101)	Nivolumab (59)		[13]
CheckMate 067 Phase 3	Hodi FS	Melanoma	Nivolumab plus Ipilimumab (314)	Nivolumab (316)	Ipilimumab (315)	[30]
CheckMate 069 Phase 2	Hodi FS	Melanoma	Nivolumab plus Ipilimumab (95)	Ipilimumab (47)		[31]
CheckMate 214 Phase 3	Motzer RJ	Renal-Cell Carcinoma	Nivolumab plus Ipilimumab (550)	Sunitinib (546)		[32]
CheckMate 227^a^ Phase 3	Hellmann MD	NSCLC	Nivolumab plus Ipilimumab (583)	Nivolumab (396)	Chemotherapy (583)	[33]
NCT02374242^b^ Phase 2	Long GV	Melanoma	Nivolumab plus Ipilimumab (35)	Nivolumab (31)		[34]
Checkmate 143^c^ Phase 1	Omuro A	Glioblastoma	Nivolumab plus Ipilimumab (10)	Nivolumab (10)		[35]

*NSCLC* non-small-cell lung cancer

^a^583, 396, and 583 patients were assigned to Nivolumab plus Ipilimumab group, Nivolumab group, and Chemotherapy group in CheckMate 227 totally. However, the difference comparison among three groups was just conducted in patients harboring at least 10 mutations per megabase

^b^NCT02374242 contained three cohorts: Cohort A (Nivolumab plus Ipilimumab, n = 35), B (Nivolumab, n = 25), and C (Nivolumab, n = 6). Among three cohorts, Cohort A and B consisted of patients with the same diagnosis and treatment. 60 patients were randomly assigned to Cohort A and B while 6 patients were selected to Cohort C. Therefore, the following analysis was based on Cohort A and B

^c^Checkmate 143 consisted of three cohorts: Cohort A (Nivolumab, n = 10), Cohort B (Nivolumab plus Ipilimumab, n = 10), and Cohort C (Nivolumab plus Ipilimumab, n = 20). 20 glioblastoma patients were 1:1 randomly assigned to Cohort A and B while 20 patients were allocated to Cohort C. Thus, the following analysis was based on Cohort A and B

### Synthesized findings

**Fig. 2:**
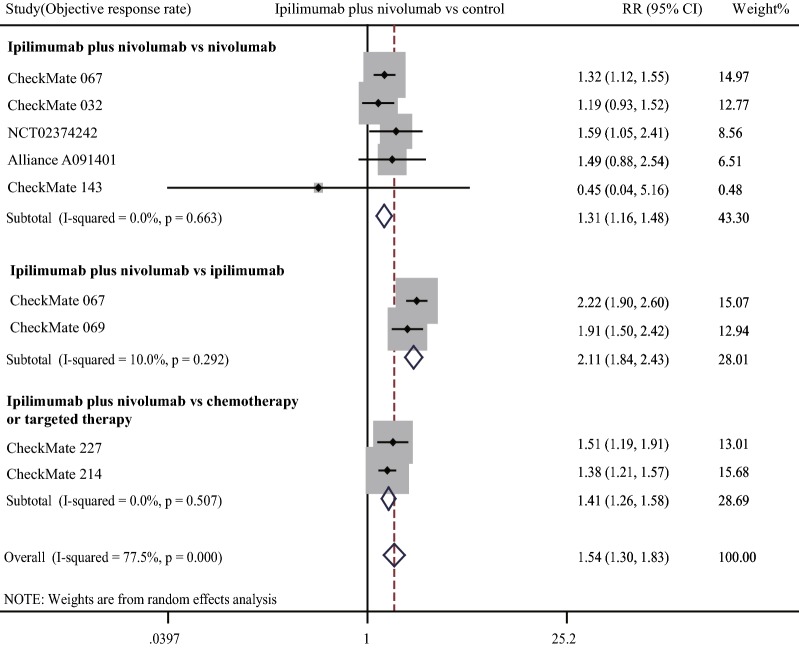
Forest plot of risk ratio (RR). Relative objective response rate (ORR) of α-CTLA-4 plus α-PD-1 combination treatment compared with other treatments. CI: confidence interval

ORR was higher in combination therapy treated patients. Our meta-analysis showed that ORR of combination therapy treated patients was significantly higher than patients undergoing other therapies (pooled RR 1.54, 95% CI 1.30–1.83) (Fig. 2). Subgroup analysis showed that combined therapy had a great advantage over α-PD-1 treatment (pooled RR 1.31, 95% CI 1.16–1.48), α-CTLA-4 treatment (pooled RR 2.11, 95% CI 1.84–2.43), and chemotherapy or targeted therapy (pooled RR 1.41, 95% CI 1.26–1.58).

**Fig. 3:**
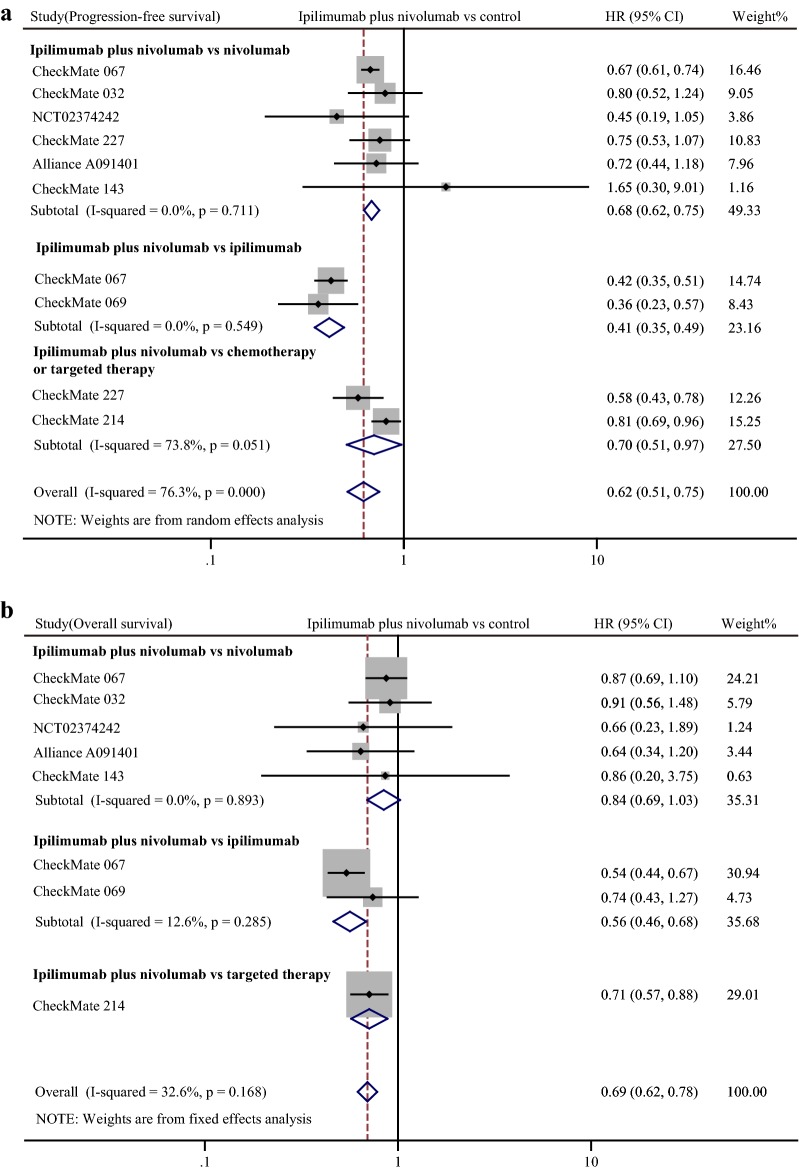
Forest plot of hazard ratio (HR). Comparison of progression-free survival (PFS) (**a**) and overall survival (OS) (**b**) between α-CTLA-4 plus α-PD-1 combination treatment and other treatments. CI: confidence interval

Outcome was better for patients undergoing combination therapy. Totally, compared with other treatments, both PFS (pooled HR 0.62, 95% CI 0.51–0.75) and OS (pooled HR 0.69, 95% CI 0.62–0.78) were improved in patients treated with combination therapy (Fig. 3). Results of subgroup analysis showed that combination treatment group had significantly improved PFS than α-PD-1 monotherapy (pooled HR 0.68, 95% CI 0.62–0.75), α-CTLA-4 monotherapy (pooled HR 0.41, 95% CI 0.35–0.49), and chemotherapy or targeted therapy (pooled HR 0.70, 95% CI 0.51–0.97) (Fig. 3a). However, results of subgroup analysis for OS showed a slightly different trend. Compared with nivolumab treatment, combination therapy had a moderate advantage in OS even without statistical significance (pooled HR 0.84, 95% CI 0.69–1.03) (Fig. 3b). In comparison with α-CTLA-4 alone, combination therapy significantly prolonged OS of patients (pooled HR 0.56, 95% CI 0.46–0.68). Limited by insufficient data, we could not conduct subgroup analysis of combination therapy vs. chemotherapy or targeted therapy (Fig. 3b). Nevertheless, available data of comparison of combination therapy vs. targeted therapy showed the advantage of combination therapy in OS (HR 0.71, 95% CI 0.57–0.88) (Fig. 3b).

**Fig. 4:**
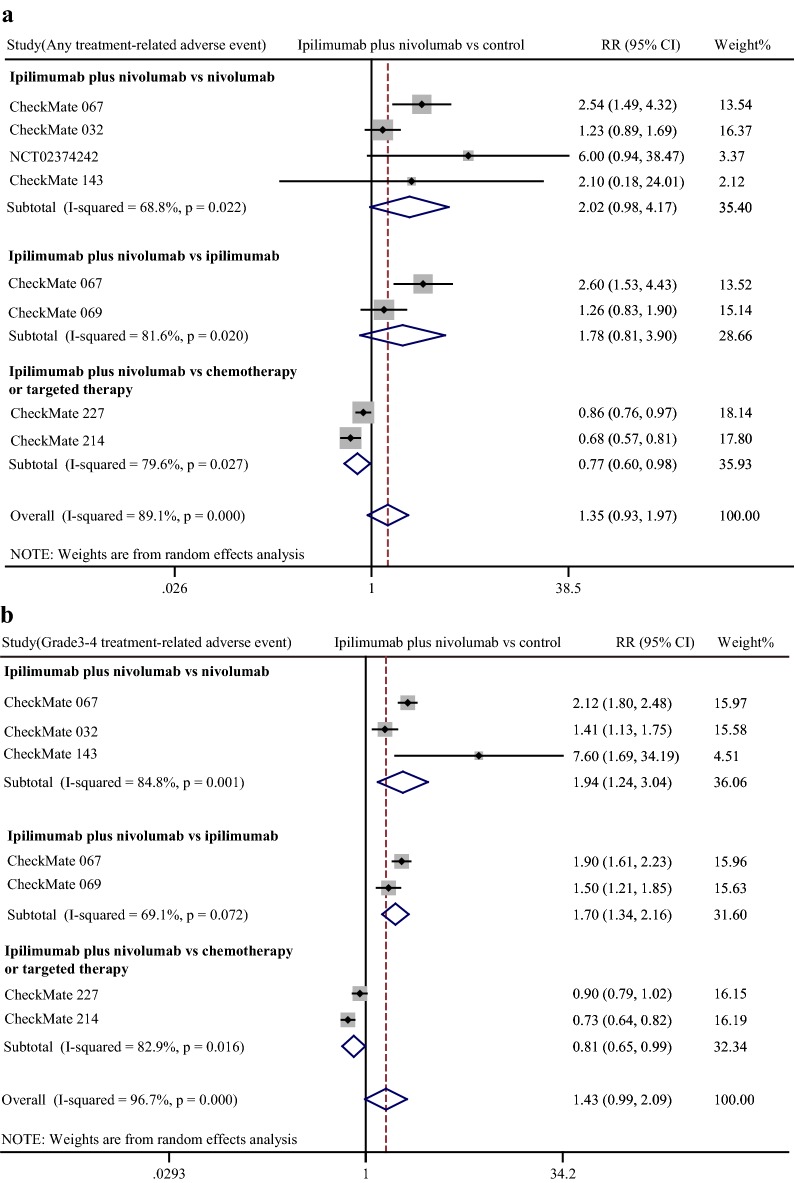
Forest plot of risk ratio (RR). Relative total treatment-related adverse event rate (**a**) and grade 3–4 treatment-related adverse event rate (**b**) of α-CTLA-4 plus α-PD-1 combination treatment compared with other treatments. CI: confidence interval

Treatment related adverse event in combination therapy. We assessed the safety of combination therapy by rate of any grade adverse event and grade 3–4 adverse event. On the whole, there was no significant difference in total adverse event rate between combination therapy and other treatments (pooled RR 1.35, 95% CI 0.93–1.97) (Fig. 4). Subgroup analysis showed that neither α-PD-1 monotherapy (pooled RR 2.02, 95% CI 0.98–4.17) nor α-CTLA-4 monotherapy (pooled RR 1.78, 95% CI 0.81–3.90) had significant advantage in total adverse event over α-PD-1 plus α-CTLA-4 treatment. Intriguingly, patients undergoing combination therapy had lower risk of total adverse event than chemotherapy or targeted therapy treated patients (pooled RR 0.77, 95% CI 0.60–0.98). Then we evaluated high-grade adverse event caused by treatment. No significant difference existed between combination treatment and other treatments in general. However, it was notable that both α-PD-1 monotherapy and α-CTLA-4 monotherapy induced less grade 3–4 adverse events than combination therapy (pooled RR 1.94, 95% CI 1.24–3.04, vs. α-PD-1 monotherapy; pooled RR 1.70, 95% CI 1.34–2.16, vs. α-CTLA-4 monotherapy), while the risk of chemotherapy or targeted therapy related adverse event was significantly higher than combination therapy (pooled RR 0.81, 95% CI 0.65–0.99).

**Fig. 5:**
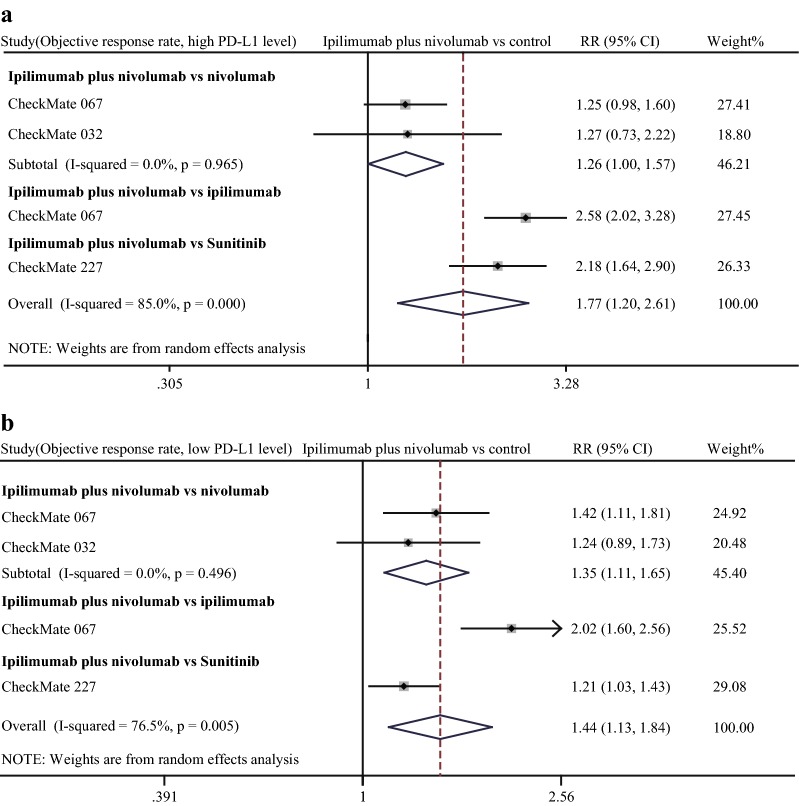
Forest plot of risk ratio (RR). Relative objective response rate (ORR) of α-CTLA-4 plus α-PD-1 combination treatment compared with other treatments in the high PD-L1 expression status (**a**) and low PD-L1 expression status (**b**). CI: confidence interval

**Fig. 6:**
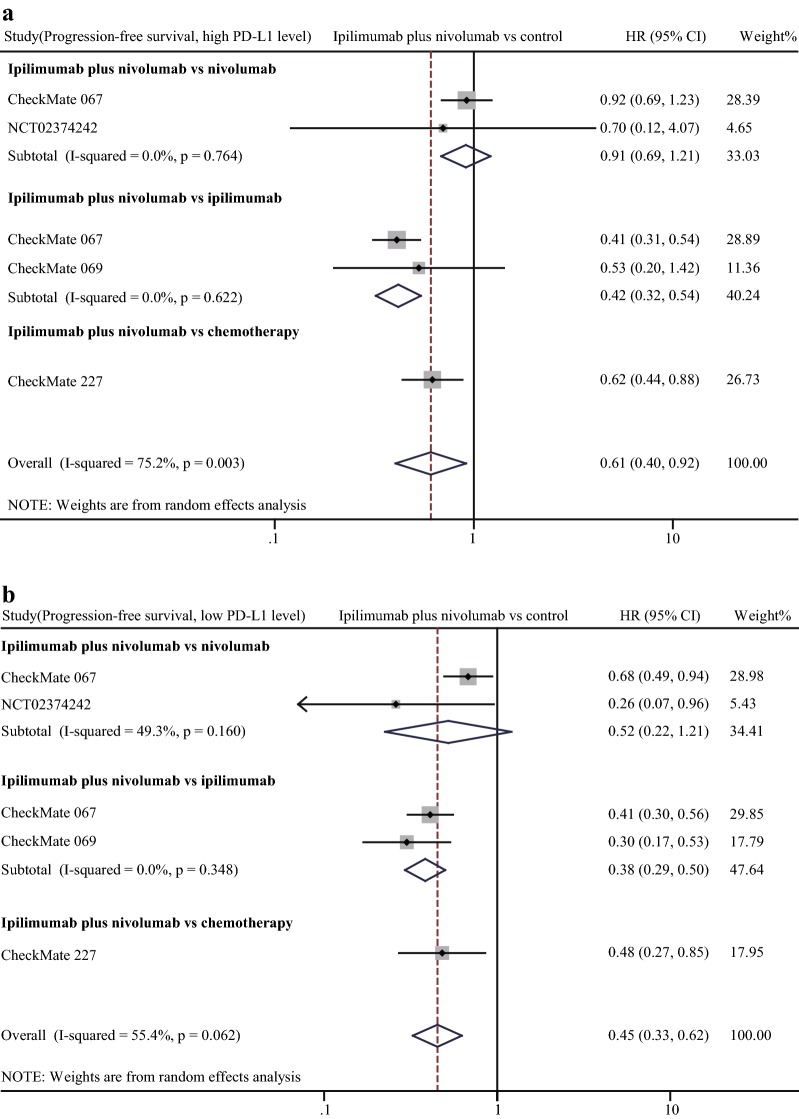
Forest plot of hazard ratio (HR). Comparison of progression-free survival (PFS) between α-CTLA-4 plus α-PD-1 combination treatment and other treatments in the high PD-L1 expression status (**a**) and low PD-L1 expression status (**b**). CI: confidence interval

**Fig. 7:**
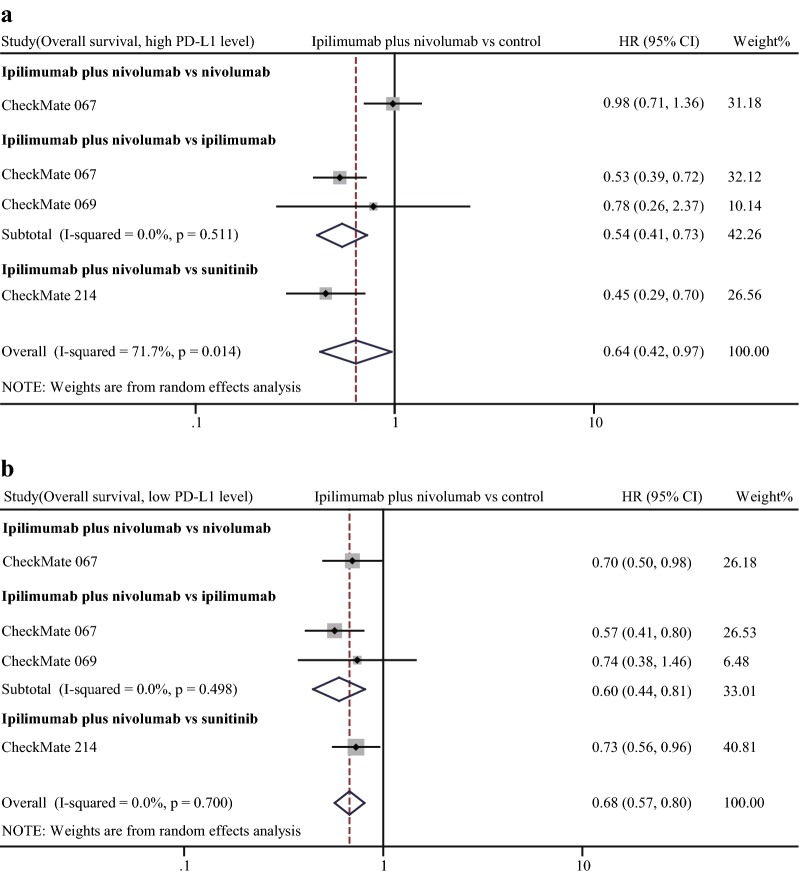
Forest of hazard ratio (HR). Comparison of overall survival (OS) between α-CTLA-4 plus α-PD-1 combination treatment and other treatments in the high PD-L1 expression status (**a**) and low PD-L1 expression status (**b**). CI: confidence interval

Efficacy of combination therapy in different PD-L1 expression statuses. In view of that PD-L1 was adopted as a predominant biomarker to screen patients for α-PD-1 treatment, we compared the efficacy of combination therapy with other treatments. In comparisons of combination therapy vs. α-CTLA-4 monotherapy and vs. chemotherapy or targeted therapy, combined therapy showed significant advantages in ORR, PFS, and OS regardless of PD-L1 expression status (Figs. 5, 6, 7). Notably, the advantages of combination therapy over α-PD-1 monotherapy were not consistent in different PD-L1 expression statuses. For patients with high PD-L1 expression (determined as ≥ 1% in the most studies), there was no significant difference between combination therapy and α-PD-1 monotherapy in ORR (pooled RR 1.26, 95% CI 1.00–1.57), PFS (pooled HR 0.91, 95% CI 0.69–1.21), OS (HR 0.98, 95% CI 0.71–1.36, data obtained from study CheckMate 067) (Figs. 5a, 6a, 7a). On the contrary, in the context of low PD-L1 expression (determined as < 1% in the most studies), patients receiving combination therapy tended to have higher response rate than α-PD-1 monotherapy (pooled RR of ORR 1.35, 95% CI 1.11–1.65) (Fig. 5b). Moreover, combination therapy treated patients tended to have a better outcome even though without reaching statistical significance (pooled HR of PFS 0.52, 95% CI 0.22–1.21) than α-PD-1 monotherapy (Fig. 6b). Despite limited data, results of CheckMate 067 showed the advantage of combination therapy over α-PD-1 monotherapy in OS (HR 0.70, 95% CI 0.50–0.98) (Fig. 7b).

### Risk of bias

According to Cochrane Collaboration’s tool for assessing risk of bias, we assessed the quality of all involved randomized controlled trials (Table 2). As a whole, 8 studies included in the meta-analysis were high-quality random controlled trials with most information at low risk of bias. Besides, we conducted a sensitivity analysis by Stata software 12.0 with metainf command. The pooled values did not changed significantly in the condition that any one study was omitted (Additional file 1: Figure S1).

**Table 2:** Cochrane Collaboration’s tool for assessing risk of bias

Study	Random sequence generation	Allocation concealment	Blinding of participants and researchers	Blinding of outcome assessment	Incomplete outcome data	Selective reporting
Alliance A091401	**√**	**√**	**×**	**×**	**√**	**√**
Checkmate 032	**√**	**√**	**×**	**√**	**√**	**√**
Checkmate 067	**√**	**√**	**√**	**√**	**√**	**√**
Checkmate 069	**√**	**√**	**√**	**√**	**√**	**√**
Checkmate 214	**√**	**√**	**×**	**×**	**√**	**√**
Checkmate 227	**√**	**√**	**×**	**×**	**√**	**√**
NCT02374242	**√**	**√**	**×**	**×**	**√**	**√**
Checkmate 143	**√**	**√**	**×**	**×**	**√**	**√**

**√**, low risk of bias; **× **, high risk of bias

## Discussion

Immune checkpoint inhibitors relieve inhibitory tumor immune microenvironment and restore T cells activity from exhausted status. Reactivated T cells could effectively recognize tumor cell-derived neoantigen and subsequently kill tumor cell. However, in clinical practice, the application of immune checkpoint inhibitor is limited by unsatisfactory response rate. Patients undergoing α-CTLA-4 or α-PD-1 monotherapy are prone to primary or adaptive resistance to immune checkpoint inhibitor. However, based on our meta-analysis, primary resistance could be overcome by α-CTLA-4 plus α-PD-1 treatment. The primary reason is that simultaneously blocked two inhibitory signaling pathways have a synergistic effect for anti-tumor immunity [19]. CTLA-4 mainly targets interaction between antigen presentation cells (APCs) and naïve T cells which interferes the expansion of T cells epitopes. Therefore, α-CTLA-4 broadens repertoire of TCR and enhances recognition of tumor associated antigen and neoantigen. Nevertheless, due to inhibitory tumor immune microenvironment, emergence of tumor specific T cells is a necessary but not sufficient condition for tumor elimination. Accompanied with accumulated tumor infiltrating lymphocytes, upregulated PD-L1 expression by inflammatory signals such as interferon-γ means deficient immune surveillance even though formation of “hot tumor”. Based on that, we speculated that α-PD-1 could substantially reduce primary resistance. Moreover, we supposed that decreased probability of adaptive resistance contributed to the improved prognosis as well. Immunoediting during the cancer progression is an important reason for adaptive resistance [20]. Broaden epitopes resulting from combination therapy could reduce the failure of recognition subclonal tumor cell-derived antigen, which provides durable and potent tumor-killing activity.

Despite all of this, the main concern of oncologists about combination therapy is the magnified risk of adverse event [21]. Our meta-analysis showed that there was no significant difference between patients received combination therapy and monotherapy in total adverse event rate. Even though monotherapy had fewer high-grade adverse event rate than combination therapy, we believed that adverse event of combination therapy was acceptable especially compared with chemotherapy or targeted therapy.

Given that PD-L1 expression strongly relates with efficacy of α-PD-1 treatment. Therefore, we investigated efficacy of combination therapy in different PD-L1 expression statuses. Notably, in the context of high PD-L1 expression, the advantage of combination therapy in efficacy over α-PD-1 monotherapy is not significant. However, in the condition of low PD-L1 expression, outcome of combination therapy was obviously better than α-PD-1 monotherapy. Our analysis suggested that for patients with high PD-L1 expression, α-PD-1 monotherapy would be a better option for minimized adverse event and medical cost. On the other hand, for patients with low PD-L1 expression, α-CTLA-4 treatment could increase patients’ sensitivity to α-PD-1 treatment. It is obvious that the combination therapy had better treatment effect than ipilimumab, chemotherapy, and targeted therapy in any PD-L1 expression condition.

In fact, other than α-CTLA-4, many interventions such as radiotherapy, oncolytic virus, and cancer vaccine have been adopted to enhance efficacy of α-PD-1 therapy [22, 23]. Due to interdependence between different anti-tumor immune stepwise events, enhanced neoantigen release, recognition, and priming/activation tumor-associated antigen or neoantigen specific T cells all indirectly promote downstream tumor-killing activity [24]. The complexity of tumor immune microenvironment suggests it is hard to completely reverse inhibitory microenvironment by a single-target therapy [25]. Therefore, combination therapy would be a promising strategy and deserves further attention.

The application of immune checkpoint inhibitors is changing the landscape of cancer therapeutics [26, 27]. In the meanwhile, from an economic point of view, it was reported the total healthcare cost of patients receiving nivolumab plus ipilimumab treatment was lower than patients undergoing nivolumab monotherapy or ipilimumab monotherapy [28]. This cost advantage of combination therapy was attributed to the lower non-drug cost due to decreased hospitalization rates after initiation treatment [28].

Some limitations still existed in our meta-analysis. Firstly, we resolved most heterogeneity by subgroup. However, we classified chemotherapy and sunitinib as one class named chemotherapy or targeted therapy, for distinguishing them from immunotherapy. Actually, this classification resulted in some heterogeneity existed in some subgroup analyses. Secondly, limited by amount of available studies, we did not analyzed efficacy of combination treatment in each specific cancer type, so our results should be carefully interpreted. Finally, our meta-analysis just included English literatures which might cause potential selection bias.

## Conclusions

Combination therapy of α-CTLA-4 and α-PD-1 had significant advantages in efficacy over monotherapy, chemotherapy and targeted therapy without significantly increased adverse event. For high PD-L1 expression patients, combination therapy did not show obviously enhanced efficacy than α-PD-1. However, for low PD-L1 expression patients, simultaneous administration of α-CTLA-4 and α-PD-1 would be an optimized strategy to acquire better clinical benefits by overcoming primary drug resistance.

## Supplementary information

**Additional file 1: Figure S1.** Sensitivity analyses to evaluate stability of meta-analysis by omitting each study. **(A)** Sensitivity analysis for pooled risk ratio (RR) of objective response rate (ORR); **(B)** sensitivity analysis for pooled hazard ratio (HR) of progression-free survival (PFS); **(C)** sensitivity analysis for pooled HR of overall survival (OS); **(D)** sensitivity analysis of pooled OR of total treatment-related adverse event; **(E)** sensitivity analysis of pooled OR of grade 3–4 treatment-related adverse event.

## Data Availability

The data supporting the conclusions of this article were retrieved from the studies according to our systematic review protocol.

## References

[1] Chen DS, Mellman I (2013). Oncology meets immunology: the cancer-immunity cycle. Immunity.

[2] Yi M, Jiao D, Xu H (2018). Biomarkers for predicting efficacy of PD-1/PD-L1 inhibitors. Mol Cancer.

[3] Marin-Acevedo JA, Dholaria B, Soyano AE (2018). Next generation of immune checkpoint therapy in cancer: new developments and challenges. J Hematol Oncol.

[4] Brunet JF, Denizot F, Luciani MF (1987). A new member of the immunoglobulin superfamily—CTLA-4. Nature.

[5] Lo B, Abdel-Motal UM (2017). Lessons from CTLA-4 deficiency and checkpoint inhibition. Curr Opin Immunol.

[6] Yi M, Yu S, Qin S (2018). Gut microbiome modulates efficacy of immune checkpoint inhibitors. J Hematol Oncol..

[7] Rowshanravan B, Halliday N, Sansom DM (2018). CTLA-4: a moving target in immunotherapy. Blood.

[8] Liu B, Song Y, Liu D (2017). Recent development in clinical applications of PD-1 and PD-L1 antibodies for cancer immunotherapy. J Hematol Oncol.

[9] Diggs LP, Hsueh EC (2017). Utility of PD-L1 immunohistochemistry assays for predicting PD-1/PD-L1 inhibitor response. Biomark Res.

[10] Patsoukis N, Brown J, Petkova V (2012). Selective effects of PD-1 on Akt and Ras pathways regulate molecular components of the cell cycle and inhibit T cell proliferation. Sci Signal.

[11] Chen X, Wang L, Li P (2018). Dual TGF-beta and PD-1 blockade synergistically enhances MAGE-A3-specific CD8(+) T cell response in esophageal squamous cell carcinoma. Int J Cancer.

[12] Tanvetyanon T, Gray JE, Antonia SJ (2017). PD-1 checkpoint blockade alone or combined PD-1 and CTLA-4 blockade as immunotherapy for lung cancer?. Expert Opin Biol Ther.

[13] Janjigian YY, Bendell J, Calvo E (2018). CheckMate-032 study: efficacy and safety of nivolumab and nivolumab plus ipilimumab in patients with metastatic esophagogastric cancer. J Clin Oncol.

[14] Hassel JC, Heinzerling L, Aberle J (2017). Combined immune checkpoint blockade (anti-PD-1/anti-CTLA-4): evaluation and management of adverse drug reactions. Cancer Treat Rev.

[15] Moher D, Liberati A, Tetzlaff J, Altman DG (2009). Preferred reporting items for systematic reviews and meta-analyses: the PRISMA statement. BMJ.

[16] Higgins JP, Altman DG, Gotzsche PC (2011). The Cochrane Collaboration’s tool for assessing risk of bias in randomised trials. BMJ.

[17] Liu Q, Li A, Yu S (2018). DACH1 antagonizes CXCL8 to repress tumorigenesis of lung adenocarcinoma and improve prognosis. J Hematol Oncol.

[18] Kiran A, Crespillo AP, Rahimi K (2017). Graphics and Statistics for Cardiology: data visualisation for meta-analysis. Heart.

[19] Swart M, Verbrugge I, Beltman JB (2016). Combination approaches with immune-checkpoint blockade in cancer therapy. Front Oncol.

[20] DuPage M, Mazumdar C, Schmidt LM, Cheung AF, Jacks T (2012). Expression of tumour-specific antigens underlies cancer immunoediting. Nature.

[21] Seidel JA, Otsuka A, Kabashima K (2018). Anti-PD-1 and Anti-CTLA-4 therapies in cancer: mechanisms of action, efficacy, and limitations. Front Oncol.

[22] Kaufman HL, Kohlhapp FJ, Zloza A (2015). Oncolytic viruses: a new class of immunotherapy drugs. Nat Rev Drug Discov.

[23] Marin-Acevedo JA, Soyano AE, Dholaria B, Knutson KL, Lou Y (2018). Cancer immunotherapy beyond immune checkpoint inhibitors. J Hematol Oncol.

[24] Yi M, Qin S, Zhao W (2018). The role of neoantigen in immune checkpoint blockade therapy. Exp Hematol Oncol.

[25] Marshall HT, Djamgoz MBA (2018). Immuno-oncology: emerging targets and combination therapies. Front Oncol..

[26] Medavaram S, Zhang Y (2018). Emerging therapies in advanced hepatocellular carcinoma. Exp Hematol Oncol.

[27] Pianko MJ, Liu Y, Bagchi S, Lesokhin AM (2017). Immune checkpoint blockade for hematologic malignancies: a review. Stem Cell Investig.

[28] Potluri R, Ranjan S, Bhandari H (2019). Healthcare cost comparison analysis of nivolumab in combination with ipilimumab versus nivolumab monotherapy and ipilimumab monotherapy in advanced melanoma. Exp Hematol Oncol.

[29] D’Angelo SP, Mahoney MR, Van Tine BA (2018). Nivolumab with or without ipilimumab treatment for metastatic sarcoma (Alliance A091401): two open-label, non-comparative, randomised, phase 2 trials. Lancet Oncol.

[30] Hodi FS, Chiarion-Sileni V, Gonzalez R (2018). Nivolumab plus ipilimumab or nivolumab alone versus ipilimumab alone in advanced melanoma (CheckMate 067): 4-year outcomes of a multicentre, randomised, phase 3 trial. Lancet Oncol.

[31] Hodi FS, Chesney J, Pavlick AC (2016). Combined nivolumab and ipilimumab versus ipilimumab alone in patients with advanced melanoma: 2-year overall survival outcomes in a multicentre, randomised, controlled, phase 2 trial. Lancet Oncol.

[32] Motzer RJ, Tannir NM, McDermott DF (2018). Nivolumab plus ipilimumab versus sunitinib in advanced renal-cell carcinoma. N Engl J Med.

[33] Hellmann MD, Ciuleanu TE, Pluzanski A (2018). Nivolumab plus ipilimumab in lung cancer with a high tumor mutational burden. N Engl J Med.

[34] Long GV, Atkinson V, Lo S (2018). Combination nivolumab and ipilimumab or nivolumab alone in melanoma brain metastases: a multicentre randomised phase 2 study. Lancet Oncol.

[35] Omuro A, Vlahovic G, Lim M (2018). Nivolumab with or without ipilimumab in patients with recurrent glioblastoma: results from exploratory phase I cohorts of CheckMate 143. Neuro Oncol.

