# Supplementary figures and images for: The efficacy and safety of combination of PD-1 and CTLA-4 inhibitors: a meta-analysis
Source: Exp Hematol Oncol. 2019 Oct 25;8:26. doi: 10.1186/s40164-019-0150-0 (PMC6815037; doi:10.1186/s40164-019-0150-0)

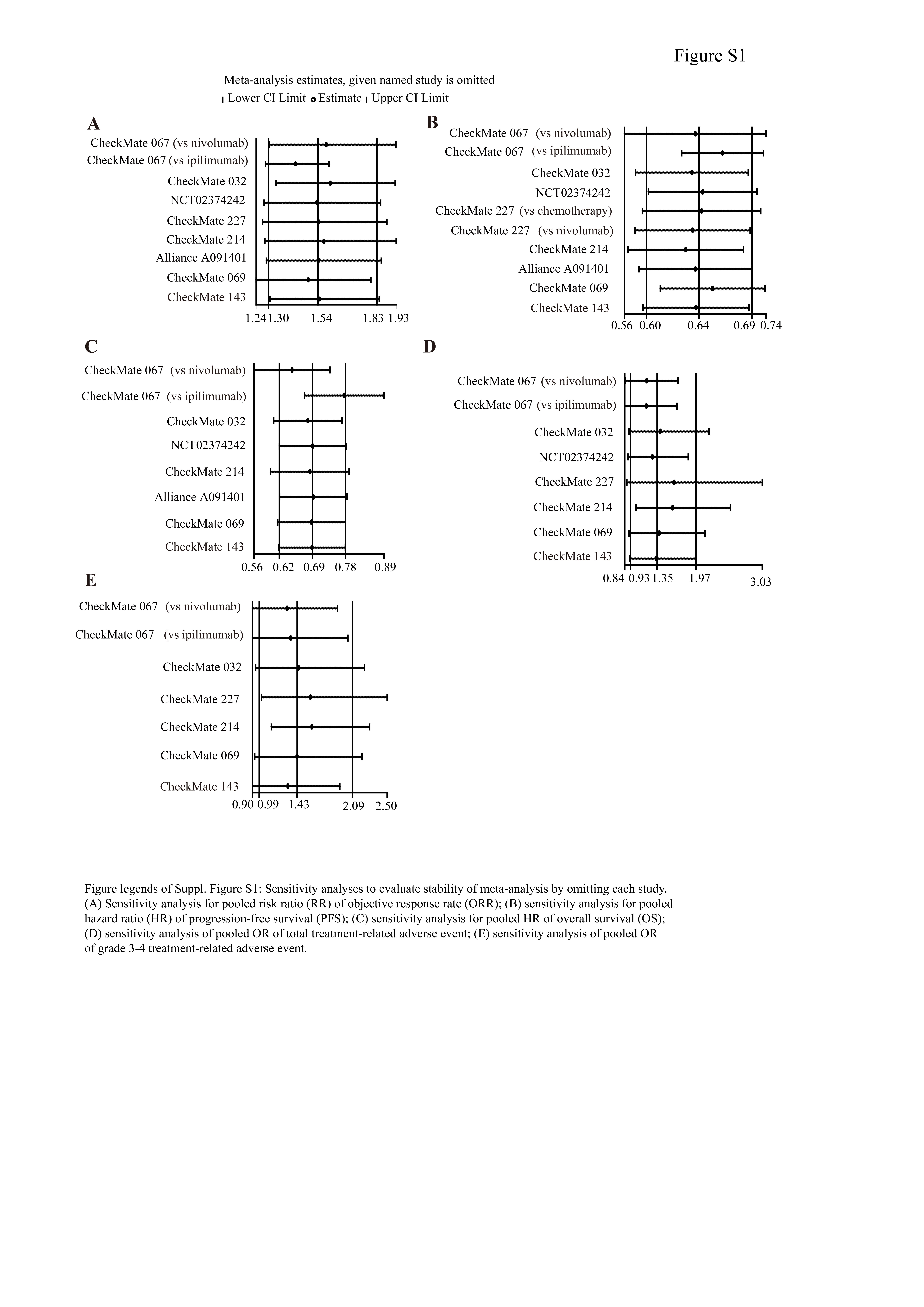

Supplement: Supplementary file 1 — Additional file 1: Figure S1. Sensitivity analyses to evaluate stability of meta-analysis by omitting each study. (A) Sensitivity analysis for pooled risk ratio (RR) of objective response rate (ORR); (B) sensitivity analysis for pooled hazard ratio (HR) of progression-free survival (PFS); (C) sensitivity analysis for pooled HR of overall survival (OS); (D) sensitivity analysis of pooled OR of total treatment-related adverse event; (E) sensitivity analysis of pooled OR of grade 3–4 treatment-related adverse event. [file 40164_2019_150_MOESM1_ESM.jpg]
